# The grand old party – a party of values?

**DOI:** 10.1186/2193-1801-3-697

**Published:** 2014-11-27

**Authors:** Patrick Mair, Thomas Rusch, Kurt Hornik

**Affiliations:** Department of Psychology WJH 968, Harvard University, 33 Kirkland St, 02138 Cambridge, MA USA; Competence Center for Empirical Research Methods, WU Vienna University of Economics and Business, Welthandelsplatz 1, 1020 Vienna, Austria; Institute for Statistics and Mathematics, WU Vienna University of Economics and Business, Welthandelsplatz 1, 1020 Vienna, Austria

**Keywords:** Multidimensional scaling, Republican party, Values, Network communities

## Abstract

In this article we explore the semantic space spanned by self-reported statements of Republican voters. Our semantic structure analysis uses multidimensional scaling and social network analysis to extract, explore, and visualize word patterns and word associations in response to the stimulus statement "I’m a Republican, because …" which were collected from the official website of the Republican Party. With psychological value theory as our backdrop, we examine the association of specific keywords within and across the statements, compute clusters of statements based on these associations, and explore common word sequences Republican voters use to characterize their political association with the Party.

## 1 Introduction

Historically, the Republican Party—often referred to as the "grand old party" (GOP)—advocated classical liberalism, paleoconservatism, and progressivism as their political ideologies. Nowadays, the GOP provides a platform for a diverse set of people including fiscal/economic conservatives, social conservatives, neoconservatives, moderates, and libertarians. Conceptually, while all these ideological branches share similar overarching (conservative) concepts, distinctions between them are based on different emphases of political values (Farmer^2006^).

The concept of "values" has received much attention in the social and behavioral sciences. The influential definition by Schwartz (1992) states that values are concepts or beliefs about desirable end states or behaviors that transcend specific situations, guide the selection or evaluation of behavior and events, and are ordered by relative importance. He identifies 10 basic values: tradition, conformity, security, power, achievement, hedonism, stimulation, self-direction, universalism and benevolence. By focusing on the structural relations between these values, Schwartz proposed that they can be arranged in a *circumplex model*. The circumplex structure means that adjacent values (e.g. tradition and conformity) tend to be positively related, and those on opposing ends (e.g., security and self-direction) are negatively related. The circumplex of values itself can be positioned in a space spanned by two dimensions (leading to four quadrants): *self-enhancement/self-transcendence* and *openness/conservation*. The first dimension is a bipolar scale opposing motives of self-interest versus motives of welfare of others. For example, instances of power and achievement are values that belong to the self-enhancement end. The second dimension opposes the wish to keep the status quo intact (conservation) with the need to pursue intellectual and emotional interests even when faced with uncertainty (openness). Examples of values related to conservation are instances of tradition, security, and conformity. An example for openness is self-direction.

Political ideology is a field in which values are particularly important and for which there exists a strong base of empirical studies (Braithwaite^1998^; Caprara and Zimbardo^2004^; Converse^1964^; Feldman^1988^,^2003^; Henry and Reyna^2007^; Jost et al^2009^; McCann^1997^; Rokeach^1973^). A substantial proportion of value studies have been conducted within the context of Schwartz’s circumplex theory (Barnea^2003^; Barnea and Schwartz^1998^; Caprara et al.^2006^; Piurko et al.^2011^; Schwartz et al.^2010^). (Schwartz et al.^2010^) investigated the relationship and congruency of core political values with basic personal values by means of a multidimensional scaling (MDS) approach. This work identified the following political values and the relationship to basic values (based on an Italian sample):

-  traditional morality (positively related to the basic values of tradition, conformity and security),
-  patriotism (security, conformity and tradition),
-  law and order (security, conformity and tradition),
-  foreign military intervention (security, conformity, tradition, power),
-  free enterprise (achievement, power),
-  equality (universalism, benevolence),
-  civil liberties (universalism, self-direction),
-  accepting immigrants (universalism, benevolence, self-direction, stimulation).

When studying data from the US, political values are often proxied by the distinction between Republicans (conservatism) and Democrats (liberalism) (McCann^2009^; Sheldon and Nichols^2009^). By shifting our focus solely to the Republican Party, we are able to tease out differences in the value structures across ideological branches. Along the lines of congruency of personal values with certain core political values (Barnea and Schwartz^1998^; Caprara et al.^2006^; Schwartz et al.^2010^), we expect Republican voters’ values to be located in the *self-enhancement/conservation* quadrant of Schwartz’ two-dimensional space, with high importance of tradition, security, achievement as well as power and conformity. This would reflect, among others, endorsement of traditional morality, law and order, patriotism, free enterprise, and foreign military intervention.

The process of performing structural value analyses based on Republican self-reports as presented in this paper poses several methodological challenges that require innovative approaches to data collection and to methodological adaptations, applications, and visualizations. First, we use a *multidimensional scaling* (MDS) variant to investigate similarities (associations) between words used in the self-reports. Second, we adapt algorithms from *social network analysis* (SNA) and graph theory to find clusters of frequently (co-)occurring word sequences. Our approach is of an exploratory nature: We investigate value representations in the unstructured data material from the self-reports, describe how they are structured and associated, and gauge how strongly they align with the relationships hypothesized Schwartz’ circumplex theory in conjunction with branches of political ideology as well as finding "mantras" often repeated word sequences in the statements.

## 2 Analysis

### 2.1 Structural exploration of word associations

The data we use in our analyses were obtained from the official website of the Republican Party (http://www.gop.com/). Up to the elections in 2012, this website hosted a section called "Republican Faces". In this section Republican voters were asked to complete the sentence "I’m a Republican, because …".

At any point in time, the Republican Faces section on the GOP website posted 180 statements as answers to the question "I am a Republican because … ". They permuted the statements dynamically every 12 hours. Consequently, we scraped the statements twice a day. After 15 days we had 5400 statements imported into the R environment for statistical computing (R Core Team^2014^) that reduced to 252 unique statements. The statements are provided as supplementary materials. We do not exactly know the composition of the sample nor how the Website maintainers selected the statements, our sample is neither fully random nor necessarily representative. Thus, although it does not lend itself to confirmatory analyses, the raw, unstructured information is well suited for data exploration.

Subsequently, we stored the data in a proper text corpus structure and performed text mining pre-processing tasks such as removing punctuations, numbers, and stop words and converting letters to lower case. We also removed the name of the corresponding person (the website provides the first name with the second name abbreviated). Using these text copora, for our first analysis we create a document-term matrix (DTM) using the tm package (Feinerer et al.^2008^). The DTM reflects a frequency table of word counts across statements. Each row in the DTM refers to a single self-report; each column to a term (keyword). The initial DTM was of dimension 252 × 734. Out of these we selected the 35 most frequent keywords which leads to a reduced DTM of dimension 252 × 35. This matrix, provided as supplementary materials, will be used in our MDS analysis. Because our data material takes the form of short and very diverse statements, the DTM is sparse which makes the structure analysis challenging. In order to scale word associations based on a sparse DTM we propose an MDS variant that is based on word co-occurrences and which we call *power gravity MDS* (PGMDS). We elaborate on this approach in the next paragraphs.

#### 2.1.1 Multidimensional scaling: SMACOF

Let us start with some general elaborations on MDS which represent the base of our gravity scaling approach. The approach we use for fitting MDS is called SMACOF (Scaling by MAjorizing a COmplicated Function) which provides a least-squares based approach to solve the MDS target function using a majorization algorithm (De Leeuw^1977^).

MDS input data are typically an *n* × *n* matrix Δ of dissimilarities with elements *δ*_*ij*_. Δ is symmetric, nonnegative, and hollow (i.e., it has zero diagonal). The problem we solve is to locate *i* = 1,…,*n* points in low-dimensional Euclidean space such that the distances between the points approximate the given dissimilarities *δ*_*ij*_ as good as possible. In other words, we want to find an *n* × *p* matrix *X* such that *d*_*ij*_(*X*) ≈ *δ*_*ij*_, where1

The index *s* = 1,…,*p* denotes the dimension of the Euclidean space. The elements of *X* are the object configurations. Hence, each object is scaled in a *p*-dimensional space such that the distances between the points in the space match the observed dissimilarities as closely as possible. The target function to be minimized in the SMACOF approach is Kruskal’s *stress*:2

with the normalization constraint3

*W* is a known symmetric *n* × *n* matrix of weights *w*_*ij*_. If all dissimilarity weights are supposed to be equal, all matrix elements are 1. Otherwise, element *w*_*ij*_ can be set to a specific value reflecting an *a priori* weight for dissimilarity *δ*_*ij*_. If some dissimilarities *δ*_*ij*_ are missing or should not be regarded in the MDS fit, *W* can be used to account for that by setting *w*_*ij*_ to 0. These elements are blanked out during the subsequent optimization process.

Let us have a closer look at the input dissimilarity matrix Δ used in equation (2). The most commonly used distance measure is the Euclidean one but various other types of input dissimilarity measures can be considered, depending on the (scale) properties of the raw data matrix (Cox and Cox^2001^). In applications in information retrieval and text mining, for instance, the cosine dissimilarity is widely used for classification and scaling applications (Chen et al.^2009^; Ye^2003^). Essentially, it reflects the angular cosine between the two vectors involved and normalizes the document length during comparison.

**Figure 1:**
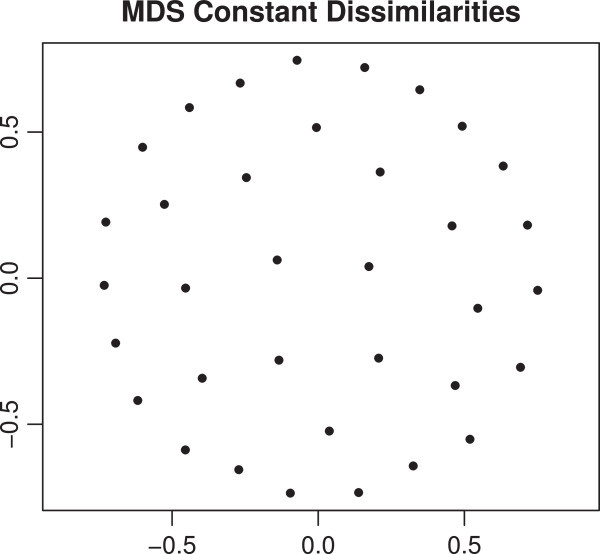
**MDS solution based on constant input dissimilarities.**

As mentioned, in our application on political values we operate on a sparse DTM. Note that such a sparse DTM setting is very common for text data and, therefore, makes our approach applicable to a wide variety of sparse and/or DTM-based MDS applications. The problem with such data is that using cosine distances (or other well-known distance measures such as Euclidean distance or Jaccard distance) lead to a so called "special MDS" solution since the dissimilarities have a very low variance: i.e. they are "almost equal". (Borg and Groenen^2005^) call it, "a special solution because of almost equal dissimilarities" (see also Buja et al.^1994^). Applying an MDS based on standard distance measures would lead to a concentric, circular representation of the configuration in the low-dimensional space that does not reveal or reproduce structures in the observed data matrix. Figure1 shows such a solution based on constant input dissimilarities. Any permutation of the labels over the points give another local minimum, so that the labels can be almost arbitrarily be assigned to the points. We solve this problem by the using a word co-occurrence based gravity approach.

#### 2.1.2 Co-Occurrence MDS

In order to formalize the gravity model we first need to elaborate on the MDS co-occurrence setting. Borg and Groenen (2005) give an overview of the use of co-occurrence data within an MDS context. In the field of text analysis, first applications of co-occurrence data trace back to He (1999) who uses the term "co-word analysis". More recent applications can be found in Van Eck and Waltman (2009) and Netzer et al. (2012).

Let *Y* be the *N* × *n* data matrix, that is, in our example, a DTM with *N* documents in the rows and *n* terms in the columns. The cells contain the frequencies obtained by counting the number of appearances of term *i* (*i* = 1,…,*n*) in document *v* (*v* = 1,…,*N*). Since we operate on counts, *y*_*vi*_ ≥ 0. Let us transform the data matrix *Y* into a co-occurrence structure by creating an indicator matrix *Y*^∗^ according to the following rule:

if *y*_*vi*_ > 0,
if *y*_*vi*_ = 0.

In order to compute the co-occurrences, that is, how often terms occur together across the whole set of documents (i.e. statements), we compute the cross-product4

The resulting symmetric co-occurrence matrix *C* is of dimension *n* × *n*. The main diagonal elements reflect the word frequencies across all statements, the off-diagonal elements how often particular words co-occur. This co-occurrence matrix is the core component of our subsequent gravity model.

#### 2.1.3 Gravity MDS

The foundation of the gravity model is Isaac Newton’s "law of universal gravitation" developed upon observing an apple fall from a tree. It was published in 1687 in his work "Philosophiae Naturalis Principia Mathematica". He states that force *F* is proportional to the product of the two masses *m*_1_ and *m*_2_, and inversely proportional to the square of the distance between them (denoted as *r*^2^). Formally, it is expressed as5

with *G* as the gravitational constant.

The gravity model is not limited to the area of physics. In fact, Haynes and Fotheringham (1988) present a wide range of gravity applications from different fields of research. All variants and applications use the formula above as the basic expression to describe certain interactions, proximities, behaviors, and transactional flows between variables that, mathematically, are formulated through Newton’s gravity expression.

In our specific application, the interaction between words *i* and *j*, expressed through word co-occurrence *c*_*ij*_, can be formulated in analogy to the gravity model in (5). Formally, this can be expressed as6

with *c*_*i*+_ as the row margins and *c*_ + *j*_ as the column margins of *C*. The unknown components in this gravity equation are the dissimilarities *δ*_*ij*_. Hence, we transform (6) as follows:7

Note that we introduce the superscript (*G*) to denote that we are referring to gravity dissimilarities. This specification leads to a dissimilarity matrix Δ^(*G*)^ that acts as MDS input matrix.

There is one more thing to consider here. If *C* is sparse, as in our application, we have instances with no overlap between the terms, i.e. *c*_*ij*_ = 0. Consequently, goes to infinity which is not feasible within an MDS context. In order to perform the minimization as given in (2), specifying the following weight structure in *W* does the trick:

- *w*_*ij*_ = 0 if *c*_*ij*_ = 0,
- *w*_*ij*_ = 1 if *c*_*ij*_ > 0.

As a consequence, during the optimization the cells with 0 weights are blanked out.

Still, by applying these pre-processing steps, it can happen that in a resulting MDS solution there is only little structure visible in the configuration plot. As a solution, we can emphasize larger distances in the gravity dissimilarity matrix Δ^(*G*)^, and, thus, bring more structure into the MDS solution by the following transformation. We can take, as given in (7), to the power of *λ*, that is,8

This leads us to the *power gravity model* and, if used in conjunction with MDS, it leads to the *power gravity multidimensional scaling* (PGMDS) model. The exponent *λ* needs to be chosen *ad hoc*: The larger *λ*, the stronger the emphasis on large dissimilarities. If *λ* = 1 we end up with an ordinary gravity MDS. For a *λ* ∈ [-*∞*,1] the effect would be to shrink large dissimilarities and increase small dissimilarities which might also be desired for other applications. There is a trade-off between the structure determined by *λ* and the goodness-of-fit as quantified by the stress value: The more structure we create, the higher the stress value. In our analysis we choose *λ* = 2 which provides a good trade-off between structure and goodness-of-fit.

### 2.2 Network communities

The PGMDS approach operates on an isolated, context-free level: The information about the particular word sequence is lost by the reduction of the data to a DTM. In this section we analyze the words in context: as word sequences used in the self-reports. This task is accomplished by adapting approaches from *social network analysis* (SNA).

One goal of this particular analysis is to see whether we are able to discover *Republican mantras*, that is, frequent, prototypical sequences that often appear in statements regarding Republican values, as exemplified in various textbooks (Bates^2011^; Latour^2007^; Root^2006^; Smith^2011^) or as reported in printed, online, and TV news coverage. We will then interpret these mantras within the context of the explored value structures from the first analysis and in conjunction with ideological subgroups of the Republican party.

#### 2.2.1 Network analysis

Social Network Analysis (SNA; see Wasserman and Faust^1994^, for a comprehensive overview) originated in fields like Sociology and Education. Nowadays, SNA has a wide range of applications whenever the aim is to study relational data. One challenge in modern SNA is the exploration of large-scale networks.

As mentioned above, our network approach operates on word sequences. The starting point is the re-organization of each single self-report in terms of pairs (*collocations*) of subsequent words (stop words are again removed). For instance, the sequence "stand for freedom and limited government" becomes *(stand → freedom)*, *(freedom → limited)*, *(limited → government)*. Each of these words defines a node (or vertex) in a network and the directed edges are defined by the collocations and the implicit word order. We thus end up with a directed graph where each edge connects the first and second word in a collocation. The weight of the edges results directly from the frequencies of the collocations across all statements. This setting enables us to gauge typical sequences as paths of a series of collocations which, in turn, allows us to identify subgraphs of densely connected nodes (*word communities*).

Technically, the network analysis consists of two parts: First, the basic network plot is produced using the Fruchterman-Reingold algorithm (Fruchterman and Reingold^1991^), a standard SNA scaling algorithm in order to optimize the position of the nodes in the network plot. Second, we group sets of nodes into clusters according to the connection density. An efficient algorithm to determine communities in large scale directed networks is the Walktrap algorithm (Pons and Latapy^2005^), an approach based on random walks. Both approaches are implemented in the R package igraph (Csardi and Nepusz^2006^) which we use for this second part of our structural analyses.

## 3 Results

### 3.1 MDS Results: republican values

**Figure 2:**
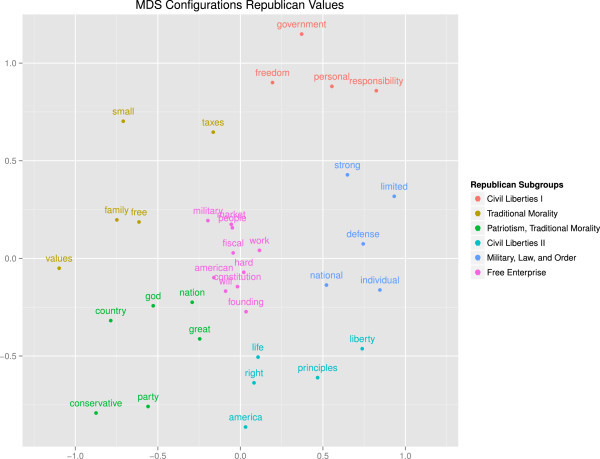
**Word configuration plot resulting from PGMDS showing the associations between keywords in self-reports and value clusters.**

The PGMDS solution is computed by feeding the gravity dissimilarity matrix into the R package smacof (De Leeuw and Mair^2009^). As a main output we get the resulting configurations in a two-dimensional space, reflecting word associations. The corresponding configuration plot and a fan dendrogram based on a post MDS hierarchical cluster analysis (using Ward’s criterion) are given in Figures2 and3.

**Figure 3:**
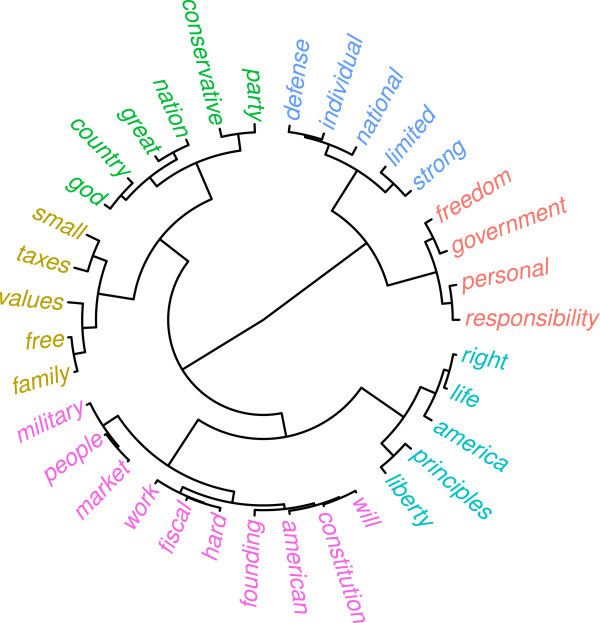
**Fan dendrogram resulting from post MDS cluster analysis.**

The clustering results in conjunction with the PGMDS configurations provide a detailed insight into word associations and provide clues into the values that inform the ideological subgroups that make up the GOP. For example, looking at the lower left quadrant of the configuration we find a cluster of frequently co-occurring words: *conservative*, *god*, *nation*, and *country*. This cluster is mostly congruent with the core political values of "traditional morality" and "patriotism" as evidenced by statements like " …I am proud to be a citizen of the greatest nation on earth and I show this by my years of military service – God bless America!" or " …the GOP best helps my ability to fulfill my obligations to god, family, and country". Another traditional morality cluster occurs on the left hand side of the plot with the keywords *free*, *family*, and *values*. Interestingly, *small* and *taxes* are assigned to this cluster as well due to statements like " …because I support small government, low taxes, and conservative family values". This cluster bears strong resemblance with themes of "social conservatism".

The center of the MDS space is inhabited by a cluster of words that relate to Schwartz’ core political value of "free enterprise", with the words, *market, fiscal, free, people, work, hard* frequently co-occurring. Here the cluster reflects support of a free market and the wish for fiscal discipline. Such fiscal conservatism and free market attitudes are characterized by statements such as "I believe in a free market society which enables hard work to equal success". The lower end of the cluster leans towards the patriotism/traditional morality cluster. The term *military* is often used in conjunction with economic statements even though, conceptually, it would fit better into the following cluster.

In the right hand area of the plot there appears a cluster with the sentiment that the GOP stands for strong military power and national defense, as reflected by the co-occurrence of *national*, *defense* and *strong*. This corresponds to statements by people that envision the US to be a strong military force, as reflected in " …because I’m an American citizen, I believe in a strong national defense" or " …because my family believes in strong military defense". We found no statement where the people directly addressed foreign military intervention but nevertheless, this cluster relates most directly to Schwartz’ core political value of "foreign military intervention" one of the main points of the neoconservatives agenda (Halper and Clarke^2005^).

At the top of the plot there is a cluster consisting of terms such as *freedom*, *personal*, and *responsibility*. A typical statement would be " …I believe in freedom and personal responsibility which is the foundation of our great country and the fuel that keeps us going" or " …we will always be the party of individual freedom". This cluster directly reflects the core political value of "civil liberties". The term *government* is scaled closely to *limited* and *small*. Limited/small government is the one of the core aspects of classical liberalism.

Another civil liberties cluster, which is positioned at the bottom of the plot, contains terms such as *right*, *life*, *liberty*, and *principles*. These reflect John Locke’s philosophical tradition of liberalism. In the northern periphery of the cluster we see that the term *individual* – which in the statements is strongly tied to *liberty* – is assigned to the neighbor cluster.

### 3.2 Network results: republican mantras

**Figure 4:**
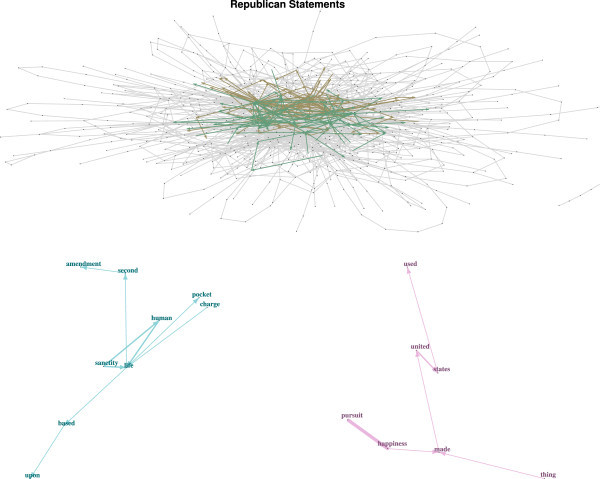
**Top panel: full network with two big communities.** Bottom panel: two small network communities.

**Figure 5:**
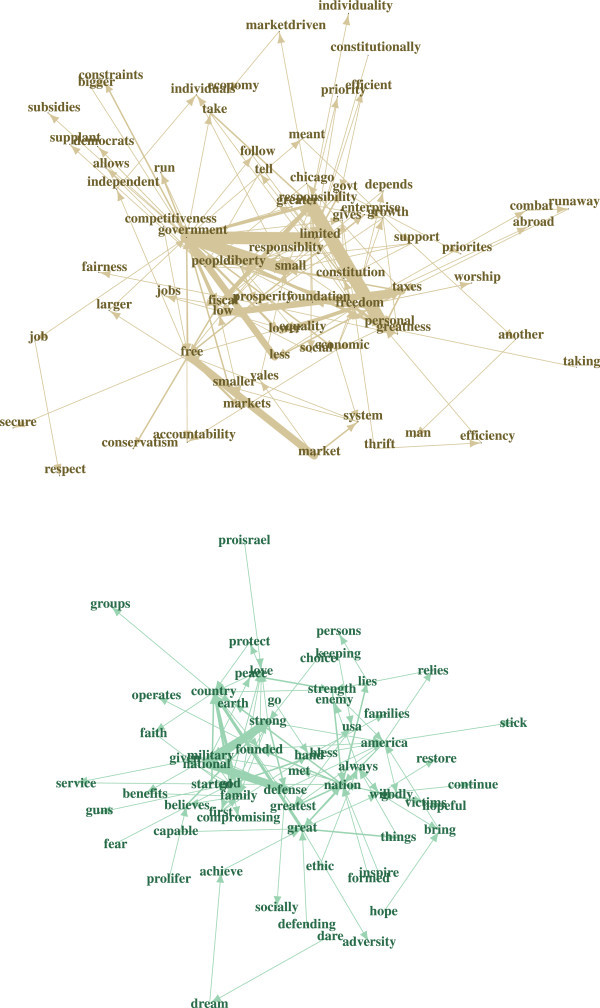
**Big network communities from Figure** 4.

By using the network approaches described above, we are able to discover 182 communities in total, of varying sizes. First, we consider the two largest communities that have 73 and 64 nodes, respectively. The top panel in Figure4 shows the full network plot with the big community overlay. For better readability the big communities with the node labels (i.e. words) are given in a separate plot (see Figure5). The bottom panel of Figure4 shows two interesting small communities.

Among the most frequent big community collocations, given in the top panel of Figure5, is *(free → market)*. This collocation, together with individual and personal achievement/responsibility, is the primary factor concerning economic prosperity as advocated by supporters of the Republican Party. The libertarian aspect of this community is represented by typical collocations such as *(limited/small → government)*, *(individual → freedom)*, *(personal → freedom)*, *(personal → responsibility)*, *(responsibility → freedom)*, and so on. This suggests that neoliberal attitudes are part of this community: " … I believe in freedom, less government, and more fiscal responsibility". The *(low → taxes)* connection is also an indicator of the presence of fiscal conservatives themes. Overall, a mantra that is quite representative here is " …I believe in individual competitiveness, the free market, limited government, and the US constitution".

The second big community in the bottom panel of Figure5 shows neoconservative topics. Halper and Clarke (2005) point out that two common neoconservative themes are: firstly, the belief deriving from religious conviction that the human condition is defined as a choice between good and evil, and, secondly, that the fundamental determinant of the relationship between states rests on military power and the willingness to use it. The word sequences in this community emphasize Christian and traditional conservative values and beliefs as being the main reason for identifying oneself with the GOP. The religious component in the definition above is evidenced by the high centrality of *god*, and the military component by the collocation *strong* and *military* as well as *national* and *defense*: " … I am proud to be a citizen of the greatest nation on earth and I show this by my years of military service – God bless America" or " …I am pro-Israel, I love our constitution as it was founded – I love freedom, God, and guns" or " …I believe in God and the Republicans’ fight for the founders’ ideals as a Christian nation" exemplify neoconservative and social conservative mantras and values of this community.As mentioned above, two interesting small communities are extracted from the network as well. The first small community given in the bottom left panel of Figure4 extends the neoconservative and social conservative aspects of the corresponding big community. The mantras "sanctity of human life" and "second amendment" are the core of this community. They are reflected by prototypical statements like " …The Republican Party represents my values on the sanctity of life, the Second Amendment, and a strong national defense" which also shows nicely the overlap with neoconservative topics as given in the second big community.

The second small community contains the mantra "pursuit of happiness" which is a rather general Republican mantra. " …The right of life, liberty, and the pursuit of happiness made the United States great and will sustain us into the future" is a representative statement in this community. Other "pursuit of happiness" statements overlap with both the big communities and the first small community. Using such a network based community finding approach, additional communities can be extracted easily.

## 4 Discussion

From a methodological point of view we present novel exploratory ways to scale and visualize word associations and to group word sequences into communities. Note that these approaches are not limited to political ideologies but can be applied to any kind of short texts. Having an environment like R which offers a wide range of tools and packages to scrape (text) data from the Web and corresponding packages to discover structural forms, gives researchers vast opportunities to perform structural text analysis using scaling methods such as MDS and SNA.

In the following paragraphs we discuss a few important topics related to MDS and possible extensions. MDS algorithms typically end up in a local minimum. The smacof package uses by default a classical scaling solution as starting configuration. Users should nevertheless explore stress values for multiple random starts and pick the configuration with the lowest stress value (see Borg and Groenen^2005^, p. 277). In our application the classical scaling starting solution leads to the lowest stress value.

Another interesting property in MDS is the possibility to compute confidence regions. For parametric confidence regions maximum likelihood based MDS models with underlying multivariate normal distributions can be considered (Ramsay^1982^). This is outside the SMACOF framework however. More modern approaches, not implemented in the smacof package yet, use bootstrap approaches to determine confidence regions (Jacoby and Armstrong^2014^). The only option provided in smacof so far is a jackknife based resampling with Procrustes matching to explore the stability of the configurations.

Other scaling techniques related to MDS (see Cox and Cox^2001^ for a discussion) are correspondence analysis (CA; see^1984^ and corresponding extensions (Gifi^1990^). In (sparse) DTMs we can have the case that single words have relatively high frequencies (compared to the remaining words). In our application, the word "hard" would be such an example. The resulting map is then "dominated" by these words since the scaling is based on *χ*^2^-distances. These words are then located far off in the plot and the remaining words are highly condensed with no visible structure. In addition, CA techniques based on singular value decomposition do not really provide opportunities to perform structural computations as our PGMDS approach.

Another interesting methodological challenge in MDS and SNA models is to incorporate covariate information. Unfortunately, we do not have any supplementary covariates in our data. If we did, the simplest way to explore the effect of categorical covariates would be to fit an MDS and SNA model for each factor level individually and compare the solutions. Within an MDS context one could apply Procrustes rotation (see e.g. Borg and Groenen^2005^, Chapter 20) to rescale the configurations such that they match as good as possible and make them comparable. For metric covariates MDS biplots (Greenacre^2010^ Chapter 4) can be used to scale the covariates on top of the configuration plot.

Overall, our analysis based on self-reports of Republican supporters highlights the diversity of values and ideology present in the modern day Republican Party and quantifies the prevalence of certain themes as well as the associations between them. Based on word co-occurrences and word sequences we are able to recover well-known psychological value structures and to identify a number of ideological branches, such as libertarianism, neoconservatism, social conservatism, and fiscal conservatism. Interestingly, it seems that one group that gets a lot of media attention is not directly identifiable: populist right–wing, ultra–conservative Republicans (as are present in, for example, the "tea party" movement).

Now that we completed our statistical journey through semantic spaces, can we answer the brave question raised in the title: Is the GOP a party of values? We have seen that Republican voters occupy the self-enhancement/conservation segment in the Schwartz value space, and that within this segment we have groups of supporters who emphasize different value structures congruent with their ideological attitude. Therefore we answer with a resounding: "Yes, we can!"
